# Performance Improvement for Discretely Modulated Continuous-Variable Measurement-Device-Independent Quantum Key Distribution with Imbalanced Modulation

**DOI:** 10.3390/e27020160

**Published:** 2025-02-03

**Authors:** Zehui Liu, Jiandong Bai, Fengchao Li, Yijun Li, Yan Tian, Wenyuan Liu

**Affiliations:** 1 School of Semiconductor and Physics, North University of China, Taiyuan 030051, China; liu664586445@foxmail.com (Z.L.);; 2 School of Information and Communication Engineering, North University of China, Taiyuan 030051, China

**Keywords:** quantum key distribution, imbalanced modulation, four-state discrete modulation, eight-state discrete modulation

## Abstract

The modulation mode at the transmitters plays a crucial role in the continuous-variable measurement-device-independent quantum key distribution (CV-MDI-QKD) protocol. However, in practical applications, differences in the modulation schemes between two transmitters can inevitably impact protocol performance, particularly when using discrete modulation with four-state or eight-state formats. This work primarily investigates the effect of imbalanced modulation at the transmitters on the security of the CV-MDI-QKD protocol under both symmetric and asymmetric distance scenarios. By employing imbalanced discrete modulation maps and numerical convex optimization techniques, the proposed CV-MDI-QKD protocol achieves a notably higher secret key rate and outperforms existing protocols in terms of maximum transmission distance. Specifically, simulation results demonstrate that the secret key rate and maximum transmission distance are boosted by approximately 77.77% and 24.3%, respectively, compared to the original protocol. This novel and simplified modulation method can be seamlessly implemented in existing experimental setups without requiring equipment modifications. Furthermore, it provides a practical approach to enhancing protocol performance and enabling cost-effective applications in secure quantum communication networks under real-world environments.

## 1. Introduction

Quantum key distribution (QKD), with theoretical unconditional security guaranteed by the fundamental principles of quantum mechanics rather than the complexity of mathematical computations, enables two distant legitimate parties to share a secure key string over an insecure quantum channel [1,2,3,4,5,6]. Recently, with applications in quantum communication, computing, metrology, and fundamental testing, quantum networks have become crucial in quantum information science. While significant theoretical and experimental advancements have been made over the past two decades in developing entangled quantum nodes and global quantum networks, the integration of quantum technology with 6G networks holds the potential to revolutionize communication. The enhanced security, accelerated transmission speeds, and expanded network capacity of the quantum internet are expected to drive the development of a diverse range of innovative applications and services [7,8]. At the same time, the twin-field quantum key distribution (TF-QKD) protocol has also received widespread attention [9,10]. At present, QKD protocols are primarily categorized into discrete-variable quantum key distribution (DV-QKD) and continuous variable quantum key distribution (CV-QKD). In CV-QKD protocol, key information is encoded into a continuous-spectrum quantum observable, such as the amplitude or phase quadrature of the light field. The security of CV-QKD protocols has been theoretically demonstrated against coherent attacks under ideal assumptions and experimentally implemented through the procedure of parameter estimation, data reconciliation, and privacy amplification. Due to its high compatibility with existing classical telecommunication systems, relatively simpler and low-cost practical implementations, and the ability to achieve a high secure key rate over metropolitan distances, CV-QKD has garnered extensive attention. Over the past two decades, significant theoretical and experimental advancements have been made in this field. Furthermore, discrete modulation schemes have been shown to achieve a relatively higher secret key rate compared to the traditional Gaussian modulation in CV-QKD, owing to improved reconciliation efficiency, particularly in long-distance transmission scenarios [11,12,13,14,15,16,17,18,19,20,21,22,23].

The CV-QKD protocol, employing either Gaussian modulation or discrete modulation, has been proven to be unconditionally secure against possible attacks under certain ideal assumptions [24,25,26,27,28,29,30,31,32,33,34,35,36,37,38,39,40,41,42]. However, in practical implementations, discrepancies between the theoretical ideal model and real-world devices introduce non-negligible gaps, potentially leading to security vulnerabilities. These loopholes can be exploited by an eavesdropper to launch unpredictable attacks, stealing information without detection. To address these vulnerabilities, the measurement-device-independent (MDI) QKD protocol was proposed [43,44,45,46,47]. In MDI-QKD, both transmitting terminals, Alice and Bob, send modulated quantum states to an untrusted third party, Charlie. Charlie performs a continuous-variable (CV) Bell-state measurement (BSM) and publicly announces the measurement results to the legitimate parties [48]. After a series of information-processing steps, a secure key is established between Alice and Bob. The MDI-QKD protocol inherently eliminates all known and unknown side-channel attacks on detectors, regardless of their nature. By leveraging this advantage, the MDI-QKD protocol facilitates the scalable deployment of CV-QKD in practical quantum communication networks.

According to previous work, all the protocols mentioned use traditional modulation methods, meaning they are based on balanced modulation techniques [49,50,51,52,53]. Symmetric Gaussian-modulated coherent states are generally used in both the theoretical analysis and experimental implementation of the CV-MDI-QKD protocol [49,54,55,56,57]. In this setup, Alice and Bob displace the quadratures of the transmitted states according to a Gaussian distribution. However, achieving perfect symmetric modulation is not feasible in realistic communication environments due to the finite range and precision of practical devices. Furthermore, imperfections in both the source and detection have been extensively studied, both theoretically and experimentally. To facilitate high-rate and low-cost applications in secure quantum communication networks, researchers have proposed discrete-modulation schemes for the CV-MDI-QKD protocol, avoiding the experimental complexity associated with Gaussian modulation. In this approach, Alice and Bob prepare M-symbol coherent states that are modulated to be equidistantly distributed in the phase space. The theoretical security of the discrete-modulation protocol has been demonstrated using convex optimization techniques, and several recent experiments have successfully implemented discrete modulation [58,59,60].

Motivated by prior work [61], we mainly extend the advantages of the discrete-modulation CV-MDI-QKD and explore the impact of imbalanced modulation on transmitters Alice and Bob in this paper. Specifically, we explore the effects of 4-phase-shift-keying(PSK) and 8-PSK on the security of CV-MDI-QKD schemes [22,23,49]. Theoretical models of the imbalanced modulation method are demonstrated under the condition of arbitrary two-mode Gaussian attacks, considering both asymmetric and symmetric distances. Based on these models, we also compare the difference of the secure key rate and secure transmission distances between imbalanced and balanced modulation. To clarify these differences, we mainly focus on the impact of specific parameters, including Shannon’s mutual information and Holevo Bound on the performance of the protocol by numerical methods considering the source and detection noise in detail. The results demonstrate that the imbalanced modulation using 4-PSK and 8-PSK increases the key rate by approximately 68.04% and 77.77%, respectively, compared to the original balanced modulation. This method of imbalanced modulation paves the way for simplifying the experimental scheme as well as significantly improving the performance of the protocol, making the protocol highly attractive for practical application. Imbalanced modulation mode also transcends the traditional discrete modulation CV-MDI-QKQ under the case of extreme asymmetric distance [55,62,63,64,65,66].

The rest of this paper is organized as follows. In Section 2, we provide a theoretical description of the CV-MDI-QKD protocol with imbalanced modulation, specifically focusing on four-state and eight-state modulations under the case of symmetric and asymmetric transmitted distances. In Section 3, we demonstrate the numeric results, including the secret key rate and maximum transmission distance for the proposed scheme. In Section 4, we summarize our results and give a discussion.

## 2. Discrete-State CV-QKD with Imbalanced Modulation

**Figure 1:**
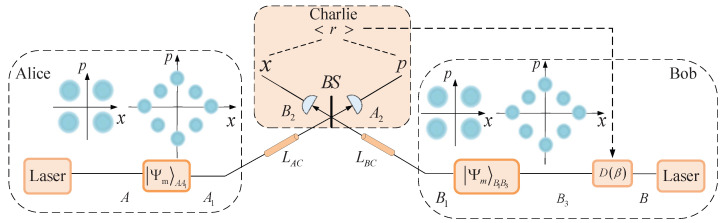
Prepare-and-measure scheme of the CV-MDI-QKD protocol. ${|\Psi_{m}\rangle }_{AA_{1}}$ and ${|\Psi_{m}\rangle }_{B_{1}B_{3}}$ are discrete modulated states prepared by Alice and Bob. Here, m=4 and m=8 are four-state and eight-state modulations. *x*—measurement results of homodyne detection for *x*-quadrature for incoming mode $B_{2}$. *p*—measurement results of homodyne detection for *p*-quadrature for incoming mode $A_{2}$. D(β)—the displacement operation of Bob. *BS*—50:50 beam splitter.

**Figure 2:**
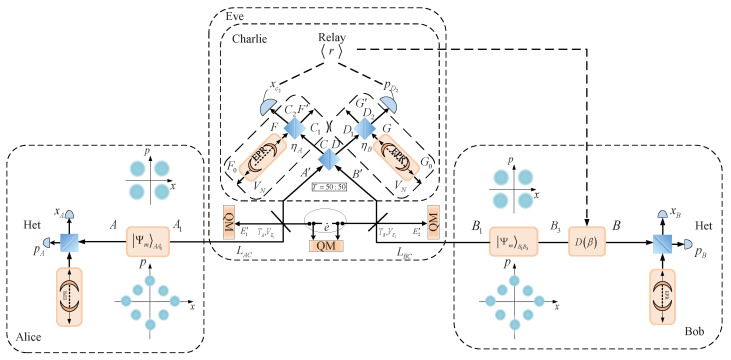
Entanglement-based scheme of CV-MDI-QKD under imbalanced modulated quantum state. Both Alice and Bob prepare the same discrete quantum state ${|\Psi_{m}\rangle }_{AA_{1}}$ and ${|\Psi_{m}\rangle }_{B_{1}B_{3}}$, except for the modulation variance, and send the other mode to an untrusted third party, Charlie, through a quantum channel with the length $L_{AC}\left(L_{BC}\right)$, respectively. Here, m=4 and m=8 are four-state and eight-state modulations. Two quantum channels and Charlie can be controlled by Eve, but Eve has no access to the apparatuses in Alice’s and Bob’s stations. The imperfection of the detectors is characterized by quantum efficiency η and the thermal state $V_{N}$ that simulates the electronic noise of the realistic detector. Het denotes the heterodyne detection. D(β)—the displacement operation of Bob. *BS*—50:50 beam splitter.

In this section, we first provide a detailed review of the CV-MDI-QKD protocol based on discrete modulation. We then examine the influence of imbalanced modulation on key parameters against a two-mode Gaussian attack in the protocol under both symmetric and asymmetric distance conditions. Furthermore, the reason for the significant improvement observed with this modulation system has also been analyzed. In general, the entanglement-based (EB) model of the CV-MDI-QKD protocol is equivalent to the standard model of prepare and measure (PM) [16,65,66]. To illustrate both models, Figure 1 and Figure 2 show the structure of the PM scheme and EB scheme based on the CV-MDI-QKD protocol, respectively. In the PM model, both Alice’s side and Bob’s side prepare the coherent state of form $|\alpha_{k}\rangle =|\alpha e^{\frac{ik2\pi }{M}}\rangle$ for some α>0, and these modulated states are transmitted to an unauthenticated third party (Charlie) through two different separated unsecured quantum channels, respectively. Charlie performs the homodyne or heterodyne detection on the incidence quantum state and announces the detection results to the Alice and Bob side simultaneously. Finally, Alice and Bob can share a common secret key over a quantum channel through a series of procedures, including parameter estimation, information reconciliation, and privacy amplification procedures. For convenience, the EB scheme is introduced to provide comprehensive proof for security analysis.

As illustrated in Figure 2, both Alice and Bob independently prepare a two-mode squeezed state with same variance (in this paper, we primarily focus on the case of different variance) and keep one of the modes on each side; the other mode is transmitted to a third party, Charlie, through the unsecured quantum channel in the EB model. Different detection methods are adopted to mode A and B, while the modes $A_{1}$ and $B_{1}$ are projected into the coherent state or squeezed state. In addition, the Einstein–Podolsky–Rosen (EPR) mode can be employed to simulate source noise and detection noise. The third relay party, Charlie, conducts continuous-variable (CV) Bell detection on the incoming modes by blending them in a balanced beam-splitter (BS) whose output ports are conjugately homodyned and the resulting outcomes are broadcast to Alice and Bob.

We begin by providing a detailed description of the prepare and measure (PM) scheme for the discrete-modulation CV-MDI-QKD protocol. The specific process is outlined as follows:

(1) Alice’s side prepares and randomly selects one of coherent states $|\rho \rangle =|{\hat{q}}_{A}+ip_{A}\rangle$, where $|\cdot \rangle$ is symbolically represented as follows:

(1) $$\{|\alpha_{k}\rangle =\alpha e^{i\frac{k\pi }{4}}\;k=0,1,2,3\;or\;k=0,1,\cdots 7\},$$

with the modulation variance of $V_{M}=2\alpha^{2}$. Then, Alice sends these coherent states to the third party Charlie through the unsecured and lossy standard quantum channel that can be controlled by the eavesdropper (Eve). At the same time, Bob performs the same operation. The third party Charlie receives two independent quantum signals and performs the measurement for the quadrature operation ${\hat{q}}_{-}=\left({\hat{q}}_{A}-ip_{B}\right)/\sqrt{2}$ and ${\hat{q}}_{+}=\left({\hat{q}}_{A}+ip_{B}\right)/\sqrt{2}$;

(2) The third party Charlie announces the results of χ, which is a complex variable consisting of ${\hat{q}}_{-}$ and ${\hat{q}}_{+}$, and χ is denoted as $\chi =\left({\hat{q}}_{-}+i{\hat{p}}_{+}\right)/\sqrt{2}$. Based on the knowledge derived from the third party, Alice and Bob can share a group of common secret keys by the process of parameter estimation, information reconciliation, and privacy amplification with the aid of an authenticated classical channel.

For the convenience of calculating the secret key rate, the equivalent entanglement-based (EB) scheme is introduced for the security analysis. Alice and Bob prepare the two-mode squeezed state ${|\Psi_{m}\rangle }_{AA_{1}}$ and ${|\Psi_{m}\rangle }_{B_{1}B_{3}}$, where *m* = 4 or *m* = 8 for the four-state and eight-state protocol, respectively. At the same time, the variances of the two-mode squeezed state of Alice and Bob are denoted as $V_{A}=V_{MA}+1$ and $V_{B}=V_{MB}+1$, respectively. The expression form of $|\Psi_{m}\rangle$ is expressed as follows:

(2) $$|\Psi_{m}\rangle =\sum_{k=0}^{m}\sqrt{\lambda_{k}}|\phi_{k}\rangle |\phi_{k}\rangle ,$$

where

(3) $$|\phi_{k}\rangle =\frac{e^{-\alpha^{2}/2}}{\sqrt{\lambda_{k}}}\sum_{n=0}^{\infty }\frac{\alpha^{nm+k}}{\sqrt{\left(nm+k\right)!}}|nm+k\rangle .$$

After deducing and integrating Equations (1)–(3), we can obtain the key parameters $\lambda_{k}$ for the four-state and eight-state modulations. For the four-state modulation [21,22], the key parameter $\lambda_{k}$ can be denoted as follows:

(4) $$\begin{array}{c} \lambda_{0,2}=\frac{1}{2e^{\alpha^{2}}}\left[\cosh\left(\alpha^{2}\right)\pm \cos\left(\alpha^{2}\right)\right]\\ \lambda_{1,3}=\frac{1}{2e^{\alpha^{2}}}\left[\sinh\left(\alpha^{2}\right)\pm \sin\left(\alpha^{2}\right)\right]. \end{array}$$

For the eight-state modulation [23], the key parameter $\lambda_{k}$ can be expressed as follows:

(5) $$\begin{array}{c} \lambda_{0,4}=\frac{1}{4e^{\alpha^{2}}}\left[\cosh\left(\alpha^{2}\right)+\cos\left(\alpha^{2}\right)\pm 2\cos\left(\frac{\alpha^{2}}{\sqrt{2}}\right)\cosh\left(\frac{\alpha^{2}}{\sqrt{2}}\right)\right]\\ \lambda_{1,5}=\frac{1}{4e^{\alpha^{2}}}\left[\sinh\left(\alpha^{2}\right)+\sin\left(\alpha^{2}\right)\pm \sqrt{2}\cos\left(\frac{\alpha^{2}}{\sqrt{2}}\right)\sinh\left(\frac{\alpha^{2}}{\sqrt{2}}\right)\pm \sqrt{2}\sin\left(\frac{\alpha^{2}}{\sqrt{2}}\right)\cosh\left(\frac{\alpha^{2}}{\sqrt{2}}\right)\right]\\ \lambda_{2,6}=\frac{1}{4e^{\alpha^{2}}}\left[\cosh\left(\alpha^{2}\right)-\cos\left(\alpha^{2}\right)\pm 2\sin\left(\frac{\alpha^{2}}{\sqrt{2}}\right)\sinh\left(\frac{\alpha^{2}}{\sqrt{2}}\right)\right]\\ \lambda_{3,7}=\frac{1}{4e^{\alpha^{2}}}\left[\sinh\left(\alpha^{2}\right)-\sin\left(\alpha^{2}\right)\mp \sqrt{2}\cos\left(\frac{\alpha^{2}}{\sqrt{2}}\right)\sinh\left(\frac{\alpha^{2}}{\sqrt{2}}\right)\pm \sqrt{2}\sin\left(\frac{\alpha^{2}}{\sqrt{2}}\right)\cosh\left(\frac{\alpha^{2}}{\sqrt{2}}\right)\right] \end{array}$$

The covariance matrix $\gamma_{AB}$ between Alice and Bob can be described as follows:

(6) $$\gamma_{AB}=\left[\begin{array}{cc} aI_{2} & c\sigma_{z}\\ c\sigma_{z} & bI_{2} \end{array}\right]=\left[\begin{array}{cc} \left(V_{MA}+1\right)I_{2} & \sqrt{T}Z_{m}\sigma_{z}\\ \sqrt{T}Z_{m}\sigma_{z} & \left[T\left(V_{MA}+1+\chi \right)\right]I_{2} \end{array}\right]$$

where $I_{2}$ is a 2×2 identity matrix, $\sigma_{z}=diag\left(1,-1\right)$. The transmission distances from Alice (Bob) to Charlie $L_{AC}$ and $L_{BC}$ are $T_{A}=10^{\frac{-\alpha L_{AC}}{10}}$ and $T_{B}=10^{\frac{-\alpha L_{BC}}{10}}$, where $T_{B}$ represents the optical fiber loss during transmission, respectively, with the parameter α=0.2 dB/km denoting the loss of optical fiber. Then, the equivalent transmission distance to a one-way protocol is $T=\frac{T_{A}g^{2}}{2}$. Here, we choose the gain of displacement $g=\sqrt{\frac{2\left(V_{B}-1\right)}{T_{B}\left(V_{B}+1\right)}}$ to minimize the equivalent excess noise; thus, we have

(7) $$\varepsilon =\varepsilon_{A}+\frac{1}{T_{A}}\left[T_{B}\left(\varepsilon_{B}-2\right)+2\right],$$

and the total channel-added noise expressed in shot-noise units is $\chi =\frac{1-T}{T}+\varepsilon$.

The secret key rate of the CV-MDI-QKD protocol with discrete modulation can be calculated as follows [44]:

(8) $$K=\beta I_{AB}-\chi_{BE},$$

where β is the reconciliation efficiency. $I_{AB}$ is the Shannon mutual information between Alice and Bob. Furthermore, it can be calculated as follows:

(9) $$I_{AB}=2\times \frac{1}{2}\log_{2}\frac{V_{AM}}{V_{AM|BM}}=\log_{2}\frac{a+1}{a+1-c^{2}/\left(b+1\right)}$$

and $\chi_{BE}$ denotes the Holevo bound between Bob and Eve. It defines the maximum information that Eve can obtain from Bob, so it is expressed as follows:

(10) $$\begin{array}{cc} \chi_{BE} & =S\left(\rho_{AB}\right)-S\left(\rho_{A}^{m_{B}}\right)\\ \; & =\sum_{i=1}^{2}G\left(\frac{\nu_{i}-1}{2}\right)-G\left(\frac{\nu_{3}-1}{2}\right) \end{array}$$

where the von Neumann entropy $G\left(x\right)=\left(x+1\right)\log_{2}\left(x+1\right)-x\log_{2}\left(x\right)$, and $S\left(\rho_{AB}\right)$ is a function of the symplectic eigenvalues $\nu_{1,2}$ of the covariance matrix $\gamma_{AB}$, given by the following:

(11) $$\nu_{1,2}^{2}=\frac{1}{2}\left[\Delta \pm \sqrt{\Delta^{2}-4D^{2}}\right]$$

with $\Delta =a^{2}+b^{2}-2c^{2}$ and $D=ab-c^{2}$, and with Eve’s condition entropy $S\left(\rho_{A}^{m_{B}}\right)$ based on Bob’s measurement result $m_{B}$ being a function of the symplectic eigenvalue $\nu_{3}$ of the covariance matrix $\gamma_{A}^{m_{B}}=aI_{2}-c\sigma_{2}{\left(bI_{2}+I_{2}\right)}^{-1}c^{T}\sigma_{2}$, which is given by the following:

(12) $$\nu_{3}=\frac{a\left(b+1\right)-c^{2}}{b+1}$$

## 3. CV-MDI-QKD Protocol for Four-State and Eight-State Imbalanced Modulations Under Symmetric Distances

**Figure 3:**
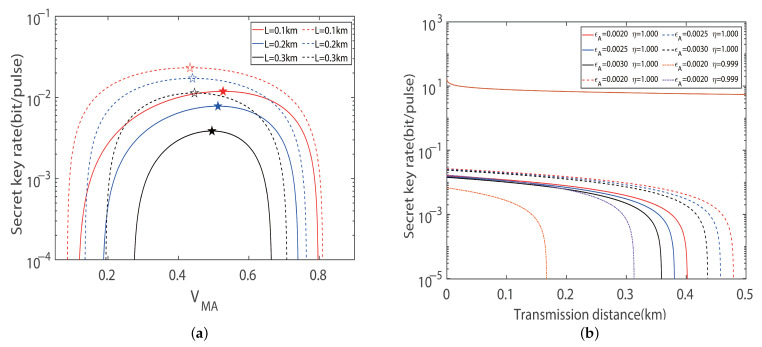
Symmetric CV-MDI-QKD with a four-state modulation. (**a**) Secret key rate versus modulation variance in the symmetric case for optimal balanced modulation (solid line) and imbalanced modulation (dashed line) variance with ideal detection, respectively. 🟉 indicates the maximum secret key rate. Here, we set the excess noise to $\varepsilon_{A}=\varepsilon_{B}=0.002$ shot noise units (SNUs). (**b**) Secret key rate versus transmission distances in the symmetric case for optimal balanced modulation (solid line) and imbalanced modulation (dashed line) variance with ideal detection, respectively. For comparing the ideal and practical detection efficiency, we set $\eta =0.999,\;v_{el}=0.001$ (orange dotted line), and $\eta =0.999,\;v_{el}=0.001$ (purple dotted line), corresponding to the optimal imbalanced and balanced modulation modes, respectively. Here, we set the reconciliation efficiency to β=0.95.

In this section, the performance of the protocol using imbalanced modulation variance with four-state and eight-state configurations is analyzed under symmetric distances $L_{AC}\;=\;L_{BC}$, where the total transmission distance, $L=L_{AC}+L_{BC}$. For the purpose of figuring out the relationship between modulation variance and the maximum secret key rate, we first investigate the dependence of the secret key rate on the modulation variance. As depicted in Figure 3a, we plot the curve about the key rate versus the modes of imbalanced and balanced modulations. The dashed lines represent the results of imbalanced modulation between Alice and Bob, which gives a higher key rate comparing the solid lines which represent the results of balanced modulation. More specifically, the optimum key rate can be obtained by performing a fine-grained search for the modulation variance $V_{MA}$ in the interval [0.1, 2] across different transmission distances. The fine-grained process is to determine the modulation variance $V_{MA}$, and then scan for the modulation variance $V_{MB}$ in the range [0.1, 2] with a step size of 0.0001 to find the maximum value. Whenever the modulation variance $V_{MA}$ changes by a step size of 0.0001, the modulation variance $V_{MB}$ repeats the above process once. Although the fine-grained search method increases the computational complexity, it can effectively improve the searching precision through advanced optimization algorithms and parallel computing technology. In terms of experimental verification, we demonstrate the performance of the fine-grained search method under different conditions, especially, with regard to its robustness in the face of noise and hardware imperfections. These results indicate that fine-grained search methods have important application value in practical quantum key distribution systems and can significantly improve the performance and robustness of the system.

Clearly, there is an optimal value of modulation variance, denoted by the star symbols, for maximizing the key rate of either balanced or imbalanced modulations. Additionally, we can see that the imbalanced modulation mode improves the key rate over the balanced modulation mode under the symmetric distances; where the total distance was set to 0.1 km, 0.2 km, and 0.3 km for comparison, the key rates are increased by 95.53%, 120.37%, and 194.09%, respectively. Based on the advantages of imbalanced modulation, we present the achievable key rates at different transmission distances for different excess noise in Figure 3b. The upper solid line is the Pirandola–Laurenza–Ottaviani–Banchi bound, which denotes the maximum secret key rate achievable in a repeater-less and lossy channel system. The key rate has been taken as the optimum value by adopting the optimal modulation variance. Under an excess noise setting of 0.002, the maximum transmission distances can be further improved by 20.27% if the imbalanced modulation is adopted to the scheme of the protocol for the four-state configuration. At the same transmission distance, the dashed-line representing the imbalanced modulation is always higher than the solid line representing the balanced modulation. In addition, the balanced modulation mode is a sub-aggregate of the imbalanced modulation mode with respect to the searching method.

**Figure 4:**
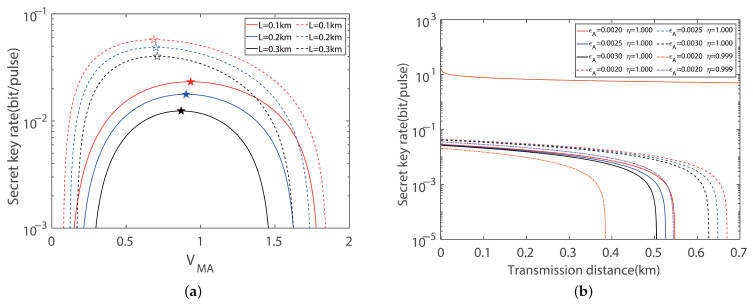
Symmetric CV-MDI-QKD with eight-state modulation. (**a**) Secret key rate versus modulation variance in the symmetric case for optimal balanced modulation variance (solid line) and imbalanced modulation (dashed line) variance with ideal detection, respectively. 🟉 indicates the maximum secret key rate. Here, we set the excess noise to $\varepsilon_{A}=\varepsilon_{B}=0.002$ shot noise units (SNUs). (**b**) Secret key rate versus transmission distances in the symmetric case for optimal balanced modulation (solid line) and imbalanced modulation (dashed line) variance with ideal detection, respectively. For comparing the ideal and practical detection efficiency, we set $\eta =0.999,\;v_{el}=0.001$ (orange dotted line), and $\eta =0.999,\;v_{el}=0.001$ (purple dotted line), corresponding to the optimal imbalanced and balanced modulation modes, respectively. Here, we set the reconciliation efficiency to β=0.95.

In order to further investigate the efficiency of the imbalanced modulation mode, the performance of the CV-MDI-QKD protocol with an eight-state configuration is demonstrated in Figure 4a,b. To maximize the key rate of the protocol, we perform fine-grained global optimization for the modulation variance, which can result in the optimal key rate. For the total transmission distance of 0.3 km, we achieve a key rate of 0.0401 bits/pulse, which is 223.19% higher than the balanced modulation mode and 253.97% higher than the protocol with a four-state configuration. With the increase in the total distance, the key rate decrease correspondingly. We observe that the key rate and total distance under the case of imbalanced modulation is significantly higher than that of the balanced modulation. Based on Figure 3 and Figure 4, the result proves that adopting the imbalanced modulation can exhibit superior performance, exceeding the traditional method of modulation.

**Figure 5:**
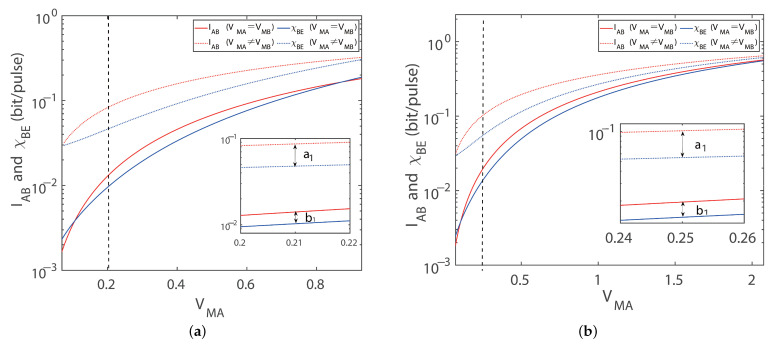
$I_{AB}$ and $\chi_{BE}$ for symmetric CV-MDI-QKD with four-state and eight-state modulations. (**a**) Shannon mutual information and $\chi_{BE}$ versus modulation variance in the four-state symmetric case for optimal balanced modulation (solid line) and imbalanced modulation variance (dashed line) with ideal detection, respectively. (**b**) Shannon mutual information and $\chi_{BE}$ versus modulation variance in the eight-state symmetric case for optimal balanced modulation (solid line) and imbalanced modulation variance (dashed line) with ideal detection, respectively. Here, we set the reconciliation efficiency to β=0.95 and the excess noise $\varepsilon_{A}=\varepsilon_{B}=0.002$ shot noise units (SNUs).

For the sake of clarifying the reasons for the advantages of imbalanced modulation, we give the simulation results for the Shannon mutual information ($I_{AB}$) and Holevo bound ($\chi_{BE}$) with the protocol for the four-state modulation. As shown in Figure 5a, by varying the modulation, the simulation results indicate that both $I_{AB}$ and $\chi_{BE}$ are correspondingly increasing under the same key parameters, including excess noise and imperfect detection efficiency. The gap denoting the key rate between $I_{AB}$ and $\chi_{BE}$ decreases gradually with the increase in the transmission distance. Notice that the difference between $I_{AB}$ and $\chi_{BE}$ under the imbalanced modulation is higher than the balanced modulation in spite the fact that both $I_{AB}$ and $\chi_{BE}$ are increasing in comparison with the balanced modulation mode. Following a similar procedure, we can also obtain the same results in the protocol with an eight-state configuration, as shown in Figure 5b. The advantage of the imbalanced modulation mode may pave the way for improving the performance of the protocol without adding extra modulation equipment. The finite size effect and composedly secure analysis can be calculated by adopting the same modulation method in future works.

## 4. CV-MDI-QKD Protocol for Four-State and Eight-State Imbalanced Modulations Under Asymmetric Distances

**Figure 6:**
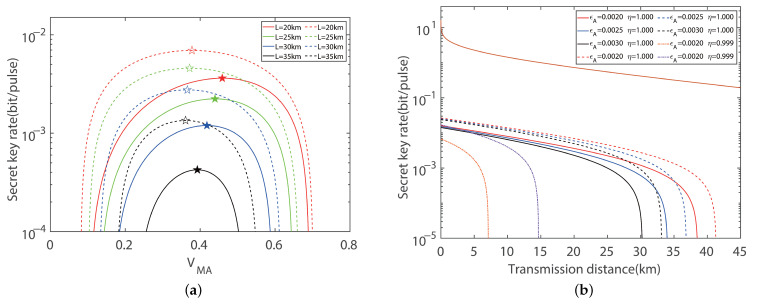
Extreme asymmetric CV-MDI-QKD with a four-state modulation. (**a**) Secret key rate versus modulation variance in the extreme asymmetric case for optimal balanced modulation (solid line) and imbalanced modulation (dashed line) variance with ideal detection, respectively. 🟉 indicates the maximum secret key rate. Here, we set the excess noise to $\varepsilon_{A}=\varepsilon_{B}=0.002$ shot noise units (SNUs). (**b**) Secret key rate versus transmission distances in the extreme symmetric case for optimal balanced modulation (solid line) and imbalanced modulation (dashed line) variance with ideal detection, respectively. For comparing the ideal and practical detection efficiency, we set $\eta =0.999,\;v_{el}=0.001$ (orange dotted line) and $\eta =0.999,\;v_{el}=0.001$ (purple dotted line) corresponding to optimal imbalanced and balanced modulation modes, respectively. Here, we set the reconciliation efficiency to β=0.95.

After introducing the simulation model results under the case of symmetric distance, the secret key rate and transmission distance are analyzed with four-state and eight-state modulations in the extreme asymmetric case in this section. Figure 6 reveals the secret key rate of the system as a function of modulation variance and transmission distance when the channel transmittance obeys the extreme asymmetric distances. The fixed key parameters for the numeric simulation are the same as the symmetric distance.

**Figure 7:**
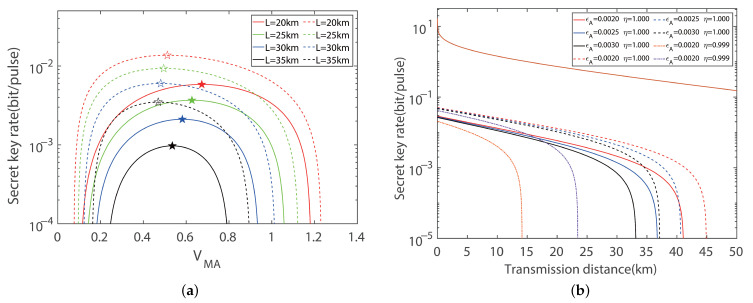
Extreme asymmetric CV-MDI-QKD with an eight-state modulation. (**a**) Secret key rate versus modulation variance in the extreme symmetric case for optimal balanced modulation (solid line) and imbalanced modulation (dashed line) variance with ideal detection, respectively. 🟉 indicates the maximum secret key rate. Here, we set the excess noise to $\varepsilon_{A}=\varepsilon_{B}=0.002$ shot noise units (SNUs). (**b**) Secret key rate versus transmission distances in the extreme asymmetric case for optimal balanced modulation (solid line) and imbalanced modulation (dashed line) with ideal detection, respectively. For comparing the ideal and practical detection efficiency, we set $\eta =0.999,\;v_{el}=0.001$ (orange dotted line), and $\eta =0.999,\;v_{el}=0.001$ (purple dotted line), corresponding to the optimal imbalanced and balanced modulation modes, respectively. Here, we set the reconciliation efficiency to β=0.95.

**Figure 8:**
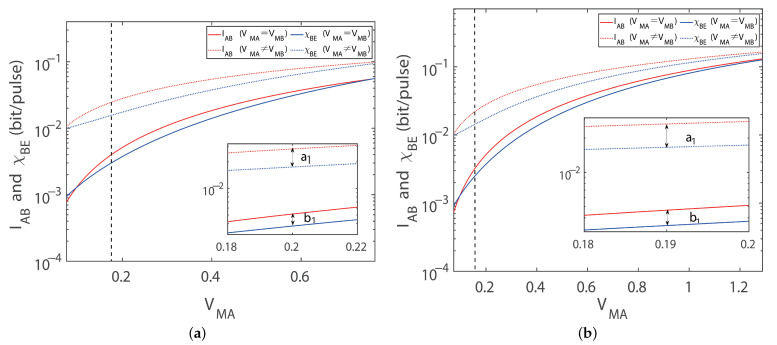
$I_{AB}$ and $\chi_{BE}$ for extreme asymmetry CV-MDI-QKD with a four-state modulation and an eight-state modulation. (**a**) Shannon mutual information and $\chi_{BE}$ versus modulation variance in the four-state extreme asymmetric case for optimal balanced modulation (solid line) imbalanced modulation (dashed line) with ideal detection, respectively. (**b**) Shannon mutual information and $\chi_{BE}$ versus modulation variance in the eight-state extreme asymmetric case for optimal balanced modulation (solid line) and imbalanced modulation (dashed line) with ideal detection, respectively. Here, we set the reconciliation efficiency to β=0.95 and the excess noise to $\varepsilon_{A}=\varepsilon_{B}=0.002$ shot noise units (SNUs).

Figure 6 depicts the secret key rate of the protocol as a function of the modulation variance in the extreme asymmetric case when the Alice and Bob utilize the balanced and imbalanced modulation mode. It is clear that there is an optimal key rate versus modulation variance regardless of imbalanced or balanced modulation, with the same results shown in Figure 3 and Figure 4. Here, the fixed key parameters for the numeric simulation results remain unchanged. For the transmission distance of 35 km, the optimal key rate of the key parameter $V_{MA}$ and $V_{MB}$ are 0.3606 and 0.3422, which result in a key rate that is 217.45% higher than that of the balanced modulation. It is obvious that the performance of the protocol with imbalanced modulation is improved compared with the balanced modulation mode. It is clear that the optimal imbalanced modulation variance is always larger than the optimal balanced modulation variance for the secret key rate. Explicitly, the total maximal transmission distances of the imbalanced modulation increase by approximately 2.91 km compared with the balanced modulation mode under certain system parameters, that is, $\epsilon_{A}=\epsilon_{B}=0.003$. The impact of the imperfections of the detectors on the performance of the protocol is also investigated in Figure 6b. To further validate the robustness and general applicability of the imbalanced modulation method, we extended the same analysis to the CV-MDI-QKD protocol with an eight-state modulation, shown in Figure 7 and Figure 8.

As mentioned above, we also analyze the reason why imbalanced modulation improves the key rate and maximum transmission distances for the extreme asymmetric distance conditions. As observed in Figure 8, with an increase in the modulation variance for both modulation modes, the difference between $I_{AB}$ and $\chi_{BE}$ grows more rapidly in the imbalanced modulation mode compared to the balanced modulation mode under extreme asymmetric distances. The details ensure the more reliable validation of the advantageous effect; the same modulation approach is implemented on the protocol with an eight-state configuration and the simulation results reveal that the imbalanced modulation mode enhances the protocol’s overall performance, including the secret key rate and transmission distances.

## 5. Discussion

As mentioned in the theoretical analysis above, the results obtained from applying the imbalanced modulation demonstrate a significant improvement in the key rate and transmission distances, with protocol performance surpassing traditional modulation techniques in both symmetric and asymmetric distance scenarios. This improvement can be attributed to the CV-MDI-QKD protocol with discrete modulation. The comparisons indicate that our approach provides a viable alternative in contexts where reliability and feasibility are critical. Despite the positive results, there are limitations to the modulation method, particularly when considering source noise and detection noise, which can be reformulated into quantum efficiency and vacuum electronic noise. Future work could address these challenges by incorporating optical phase-sensitive amplifiers (PSA) while simultaneously accounting for the finite-size effect and composable security analysis.

## 6. Conclusions

In this paper, we have theoretically investigated and analyzed the performance of the protocol with four-state and eight-state modulations, comparing imbalanced and balanced modulation methods. The security analysis demonstrates that imbalanced modulation outperforms balanced modulation under both symmetric and extreme asymmetric distance conditions. Furthermore, these simulation results provide valuable guidance for designing experimental schemes aimed at reducing cost and complexity. According to the use of the proposed advantageous modulation method, the key rate is found to be 60–70% higher than that of the previous protocol and achieves an additional transmission distance over 2.91 km compared to the traditional balanced modulation mode. The CV-MDI-QKD protocol implements a multi-party information transmission mechanism by allowing multiple transmitting ends to exchange information with the relay node (Charlie). The protocol supports the expansion of the number of transmitting terminals and converges all information to the communication center, thereby building a quantum network communication architecture. Under this framework, studying the differences in modulation variances of individual senders will help us to deeply analyze the impact of modulation imbalance on key performance indicators such as the key rate. According to the channel conditions and detector performance, the modulation variance can be dynamically adjusted to optimize the balance between the signal-to-noise ratio and the key rate. However, in practice, factors such as imperfect detection efficiency and source noise can influence the performance of the protocol. To mitigate these issues, phase-sensitive amplifiers (PSA) can be incorporated into the experimental implementation of the CV-MDI-QKD protocol.

## Data Availability

The data that support the findings of the study are available from the first author and the corresponding author upon reasonable request.
